# Bilateral Optic Disc Edema as the Presenting Sign of Brain Metastasis in Ovarian Carcinoma: A Case Report

**DOI:** 10.7759/cureus.82049

**Published:** 2025-04-10

**Authors:** Alka Tripathi, Nivedita Singh, Richa Agarwal, Neha Singh, Amit Thangjam

**Affiliations:** 1 Ophthalmology, All India Institute of Medical Sciences, Gorakhpur, IND

**Keywords:** best corrected visual acuity, brain tumors (primary or brain metastasis), metastatic ovarian cancer, optical coherence tomography (oct), papilledema

## Abstract

Brain metastases are one of the common causes of brain tumours in adults and lead to increased morbidity and mortality in cancer patients. Primary cancers such as lung, breast, and melanoma are most likely to metastasize to the brain; however, the incidence of brain metastases in ovarian cancer is low. Papilledema due to brain metastasis occurs when the tumor mass within the brain leads to increased intracranial pressure (ICP). We present an unusual case of bilateral papilledema as the earliest clinical manifestation of brain metastasis in a 48-year-old woman who had undergone treatment for ovarian carcinoma one year ago. We are trying to highlight the rare relapse of ovarian carcinoma (OC) in the form of brain metastasis within one year of therapy and presenting with ophthalmic complaints. Therefore, we emphasize detailed history taking, clinical examination, and neuroimaging of any patient so that early diagnosis and treatment can be done.

## Introduction

Papilledema is swelling of the optic nerve head that occurs due to increased pressure in the brain. It's usually bilateral and can develop for hours or weeks. The presenting symptoms are generally blurred or double vision, transient obscuration of vision, headaches, especially in the mornings and when lying down, nausea and vomiting, and pulsatile tinnitus. Causes of papilledema could be varied, such as brain tumours, cerebrospinal inflammation, idiopathic intracranial hypertension (IIH), conditions that decrease cerebrospinal fluid outflow, cerebral venous sinus thrombosis, medications such as retinoids, tetracycline antibiotics, and vitamin A derivatives [1].

Brain metastases are one of the common causes of brain tumours in adults and lead to increased morbidity and mortality in cancer patients. Primary cancers such as lung, breast, and melanoma are most likely to metastasize to the brain [2]. Ocular involvement due to brain metastases could be due to high intracranial pressure (ICP) as found in 52% of the cases in a study, tumor infiltration of the optic nerve in 17% of patients, optic nerve compression in 12%, and optic nerve inflammation in 9% of patients, and pseudopapilledema and ischaemia in rest 10% patients [3]. We present an unusual case of bilateral optic disc edema with diplopia and diminution of vision as the earliest clinical presentation of brain metastasis in a 48-year-old woman who had undergone treatment for ovarian carcinoma one year ago.

## Case presentation

**Figure 1:**
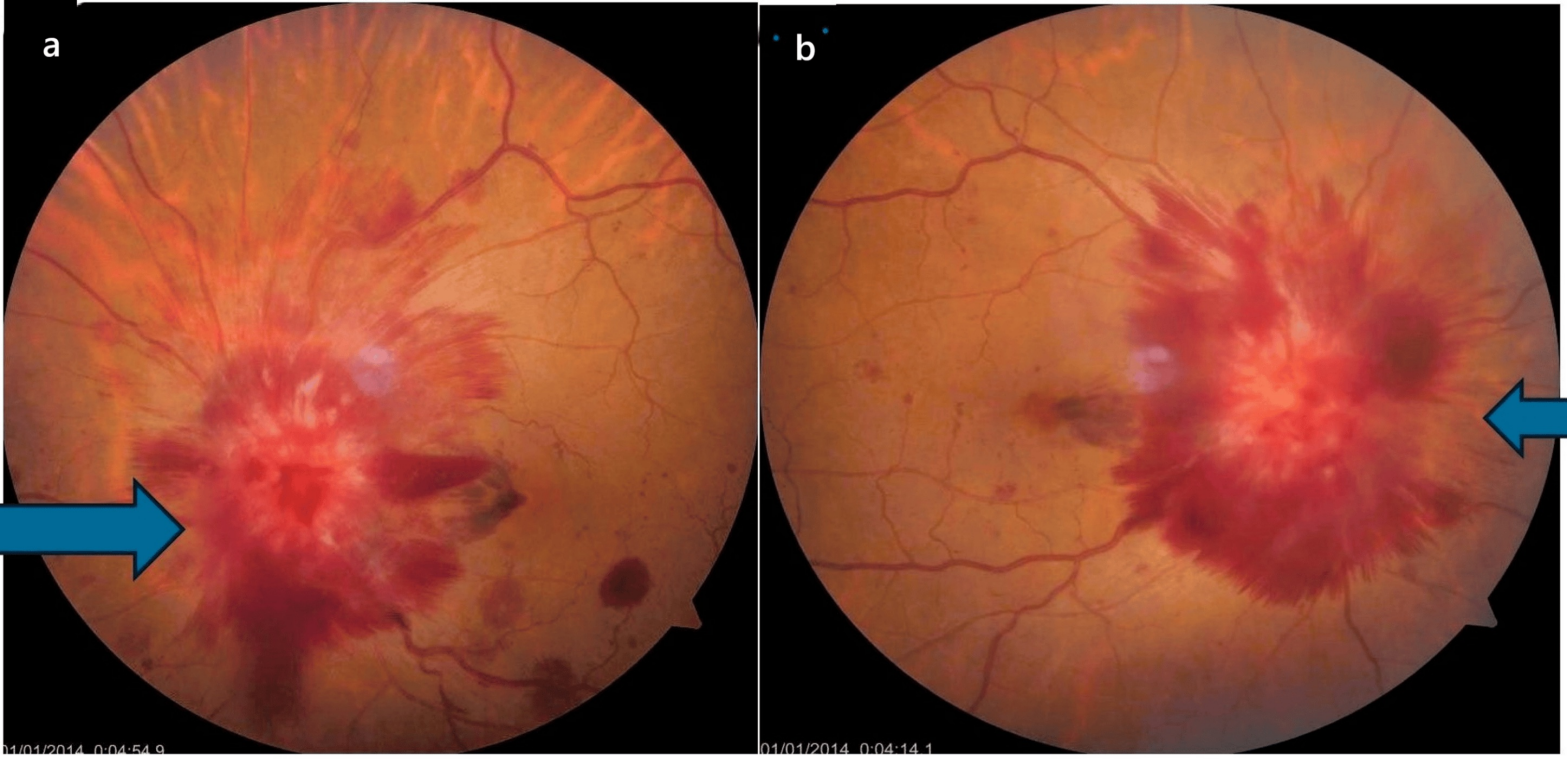
Bilateral profound disc edema. (a) OD disc and (b) OS disc. The arrows show optic disc edema. OD: oculus dexter; OS: oculus sinister

**Figure 2:**
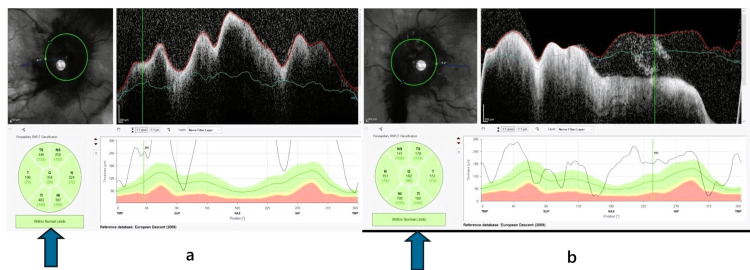
Spectral domain OCT RNFL graph showing increased thickening of the peripapillary retinal nerve fiber layer in all four quadrants (right eye more than the left). (a) OD RNFL and (b) OS RNFL. The arrows show increased RNFL thickness. OD: oculus dexter; OS: oculus sinister; OCT: optical coherence tomography; RNFL: retinal nerve fiber layer

A 48-year-old female was referred to the outpatient department of ophthalmology with diplopia and diminution of vision in both eyes for 10 days. She has been treated for carcinoma of the ovary, under remission for one and a half years, and was under the follow-up of the radiotherapy department. Routine investigations, including complete blood count (CBC), coagulation profile, liver function test, and kidney function test, were normal; however, the CA-125 level was greater than 500 U/mL. Visual acuity (VA) was oculus dexter (OD) 20/60 and oculus sinister (OS) 20/60. Slit-lamp findings were unremarkable. Extraocular movements were full and free. Fundus examination revealed bilateral pronounced optic disc swelling with disc hemorrhages surrounding the disc and absent spontaneous venous pulsations (Figures 1a, 1b). Spectral domain optical coherence tomography showed increased retinal fibre layer thickness (RNFL) in all four quadrants (Figures 2a, 2b).

**Figure 3:**
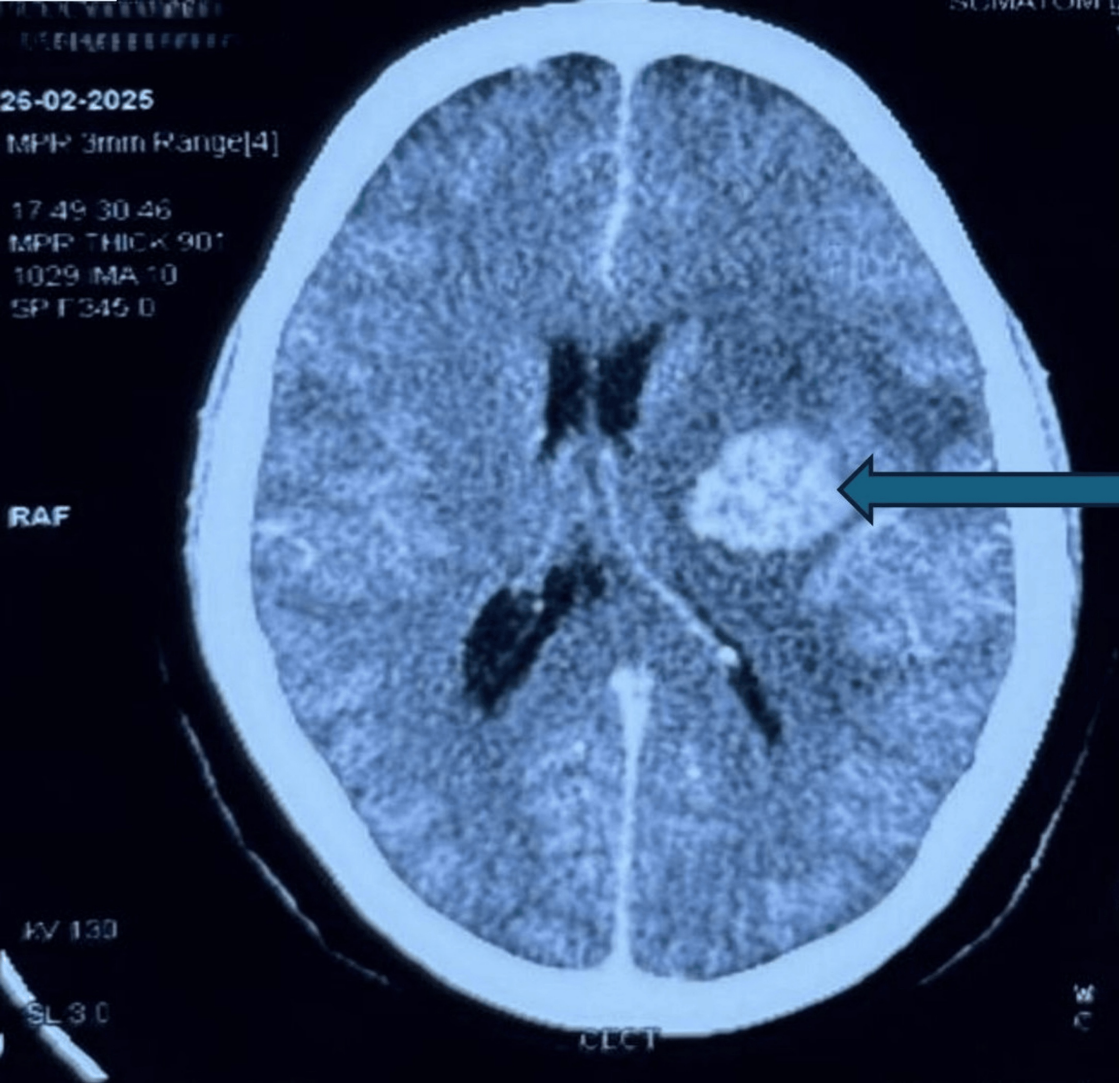
Contrast-enhanced CT scan showing an intra-axial, well-defined hyperdense lesion (HU 55-65), measuring 5.2 x 3.8 cm in left gangliocapsular region with perilesional edema. Mass effect is noted in the form of midline shift of 9.8 mm towards the right side and effacement of the left lateral ventricle.

A contrast-enhanced CT scan showed an intra-axial, well-defined hyperdense lesion in the left gangliocapsular region with perilesional edema suggestive of brain metastasis (Figure 3). The patient was prescribed carboplatin and paclitaxel drugs and adjuvant radiotherapy. Treatment was advised for this patient, but it has not been started yet. She was advised to get admitted to get the chemotherapy and radiotherapy started. The patient was also recommended to have a contrast-enhanced computed tomography (CECT) abdomen, but she did not follow-up.

## Discussion

Brain metastases are one of the common complications of cancer and account for the most common type of brain tumor. About 10-26% of cancer patients will develop brain metastases in their due course, according to a study [4]. Lung carcinoma, breast carcinoma, and melanoma are the common primary cancers leading to brain metastasis. Ovarian cancer (OC) is one of the leading causes of cancer deaths in the female reproductive system group. OC has a high tendency of local and regional recurrence as metastatic cells spread through peritoneal fluid or lymphatic drainage. Ovarian cancer (OC) is highly chemosensitive, but tumor relapses are common, typically occurring within three years after the completion of treatment. The incidence of brain metastases in ovarian cancer is around 1.34% (0.49-6.1%). Although brain metastasis is rare but the oncologists, primary care physicians, and ophthalmologists should know that it can happen months to years after treatment of OC, and subjective vision complaints as well as objective findings of the optic nerve may be the presenting sign of this unfortunate progression [5].

Metastatic cells cross the bloodstream, and after the breakdown of the blood-brain barrier, they make their way to the central nervous system [6]. Papilledema due to brain metastasis occurs when the tumor mass within the brain leads to increased intracranial pressure (ICP). The increased pressure compresses the optic nerve, reducing axoplasmic transport (the movement of materials within the nerve fibers). Axoplasmic substances collect at the lamina cribrosa (where the optic nerve fibers exit the eye), causing swelling of the optic disc [7].

Das et al. reported a case of bilateral optic disc edema as the initial clinical manifestation of brain metastasis in a woman who had undergone treatment for breast carcinoma two years ago [8]. Grixti et al. reported a case of bilateral optic disc edema due to obstructing spinal plasmacytoma without intracranial extension [9]. Kim and Pearce reported a case of unilateral optic nerve metastasis in a patient with metastatic carcinoma of the breast [10]. Cabitza et al. reported a case of a patient suffering from high-grade serous ovarian carcinoma along with a cerebellar metastasis. The metastasis and the primary tumor were surgically treated, along with adjuvant chemotherapy, and she was maintained on PARP inhibitor (PARPi) niraparib [11].

## Conclusions

The oncologists, primary care physicians, and ophthalmologists should be aware of the fact that although brain metastasis is rare in OC but it can occur anytime, even after the completion of treatment. This warrants a multidisciplinary approach, a high degree of suspicion, and a detailed past medical or surgical history of any cancer patient visiting the clinics. Visual symptoms, though rare, should prompt a complete ophthalmological assessment in such patients. The present case emphasizes that specific attention and focused research in cancer patients could lead to prompt treatment and a better prognosis. This case emphasizes that specific attention and focused research in cancer patients could lead to prompt treatment and a better prognosis. We are trying to highlight a rare relapse of OC in the form of brain metastasis within one year of therapy and presenting with ophthalmic complaints. Therefore, through this case report, we highlight the importance of detailed history taking, quarterly clinical examination, and yearly neuroimaging of cancer patients so that early diagnosis and treatment of any relapse that occurs can be done.
